# Glycoproteins in circulating immune complexes are biomarkers of patients with Indian PKDL: A study from endemic districts of West Bengal, India

**DOI:** 10.1371/journal.pone.0192302

**Published:** 2018-02-08

**Authors:** Priyank Jaiswal, Souvik Datta, Bikash Sardar, Surya Jyoti Chaudhuri, Dipankar Maji, Manab Ghosh, Bibhuti Saha, Sumi Mukhopadhyay

**Affiliations:** 1 Department of Laboratory Medicine, School of Tropical Medicine, West Bengal, India; 2 Department of Tropical Medicine, School of Tropical Medicine, Government of West Bengal, West Bengal, India; 3 Ranaghat Sub Divisional Hospital, Government of West Bengal, Nadia, West Bengal, India; 4 Department of Health & Family Welfare, Government of West Bengal, West Bengal, India; National Centre For Cell Science, INDIA

## Abstract

**Background:**

Post Kala Azar Dermal Leishmaniasis (PKDL) occurs as dermal consequence of previous Visceral Leishmaniasis (VL) infection and serves as an important reservoir for transmission of VL. Diagnosis of PKDL is often challenging for its symptomatic resemblance to other co-endemic diseases like Leprosy or Vitiligo. Parasitological examination by slit-skin smear and culture are the standard methods but lack high sensitivity. Thus, for efficient control of VL, reliable diagnostic and prognostic assay of PKDL are required.

**Objective:**

Previously, glycoproteins (9-OAcSA) have been reported as promising biomarkers of Indian VL patients. However, till date, the status of glycans in Indian PKDL patients remains unexplored. Accordingly, in this study, the glyco-profile of PKDL Circulating Immune Complexes (CICs) as compared to other cross diseases like Vitiligo and Leprosyhas been investigated. Further, a novel Glyco CIC assay has been developed for efficient Indian PKDL patient diagnosis.

**Methods/principal finding:**

In the present study, 90 PKDL patients were enrolled from 3 VL endemic districts of West Bengal during 2015–16. Glycosylation profile of isolated CICs from sera of PKDL patients were initially analyzed through gradient SDS gel electrophoresis followed by PAS silver double staining, which revealed the presence of several glycan rich PKDL specific proteins of varying molecular weights. To further characterize the glyco-profile of acid dissociated affinity purified immuno-reactive antigens present in the CICs, glycosylation was demonstrated in these purified CIC antigens by DIG glycan differentiation kit with or without glycosidase as well as neuraminidase treatment. Diagnostic evaluation of the newly developed colorimetric Glyco CIC assay through Receiver Operating Characteristic (ROC) curve analysis revealed excellent (0.99) AUC value as compared to other conventional serodiagnostic assays like PEG CIC, Parasite ELISA (IgG and IgM). Additionally, longitudinal monitoring of 18 PKDL patients further revealed its good prognostic utility.

**Conclusion:**

These results highlight the glycosylation status of CICs among Indian PKDL patients present in all the studied endemic districts of West Bengal. These PKDL biomarkers were completely absent in cross diseases like Vitiligo and Leprosy. Further, the newly developed Glyco CIC assay had an improved sensitivity of 95.6%, specificity of 99.3%, NPV of 97.1% and PPV of 98.9%.

## Introduction

Leishmaniasis is considered as one of the major parasitic disease, affecting individuals, worldwide. Residents mostly belonging to the tropical countries, with poor living conditions, serve as an easy prey for the vector sand flies, harboring the parasite of *Leishmania sp*. Currently 350 million people are at risk, worldwide, with India being one of the endemic hotspots, along with Sudan, Nepal, Brazil, Bangladesh and Ethiopia [1, 2]. In India, Bihar alone contributes to 90% of Leishmaniasis cases [3]. Moreover, Indian Leishmaniasis are profoundly manifested in two forms- namely **(i)**Visceral Leishmaniasis (VL); where patients develop characteristics of high fever, hepatosplenomegaly, anemia, loss of appetite and **(ii)** Post Kala Azar Dermal Leishmaniasis (PKDL); a dermal consequence, which occurs mostly in patients with previous VL history (10–15%), having superficial manifestations as painless dermal lesions of macular, nodular or polymorphic types [3–5]. PKDL patients are often reluctant to seek medical care as the disease results in no fatal consequences other than cosmetic complicacies, which is more of a social stigma rather than a grave problem. Considering such neglected scenario, the ongoing VL elimination program in India is targeted to reduce VL incidences to less than 1 per 10,000 population in block and sub block levels, where the upsurge of PKDL cases are posing threat to this holistic approach of elimination. Moreover, accurate case diagnosis of PKDL patients still stands as the foremost challenge for successful elimination of VL, since unattended or misdiagnosed cases of PKDL serve as a silent reservoir of *Leishmania donovani*, in VL endemic zones and play active role in the anthroponotic transmission of VL [1].

Currently, several modern tools and techniques are available for PKDL diagnosis. Parasite identification by skin slit smear is considered gold standard, but has sensitivity of only 58% for PKDL case detection as highlighted from studies in Bihar [6]. On the other hand, rk39 strip test lacks efficacy in macular PKDL case detection with only 73% sensitivity, in Indian scenario [7, 8]. In addition, the Leishmanin Skin Test (LST) for determining parasite load or culture isolation from skin biopsies is also not reliable, as it has only 54% sensitivity. Therefore, in the absence of proper diagnostics, there is likelihood that a hypopigmented macular form of PKDL can be misdiagnosed as a Vitiligo case [9–11]. Histopathological studies with patients’ skin biopsies too have very low (7–33%) sensitivity towards macular case detection [9, 12]. Further, Direct Agglutination Test (DAT), which is otherwise efficient for VL diagnosis fails in PKDL case detection [13–15]. On the other hand, high throughput confirmatory assay like microscopy and PCR/qPCR requires highly trained individuals, and has limitations in field settings [16]. Although various analytical tools for both VL and PKDL diagnosis are available, however, very few prognostic markers or biomarkers to monitor treatment response of VL/PKDL have been reported. Such biomarkers would be very useful in successfully diagnosing a cured VL/treated PKDL from an active VL/PKDL case. Since PKDL patients serve as silent parasitic reservoir of *Leishmania donovani* in VL affected regions and actively participates in the anthroponotic VL transmission [1], hence there exists an urgent need for identification of novel biomarkers and the development of an accurate diagnostic and prognostic assay.

Several studies have previously confirmed the diagnostic utility of 9-*O* acetylated sialoglycoproteins among patients with Indian VL [17–21]. Moreover, 9-*O* acetylated sialoglycoproteins were reported to have high sensitivity and specificity for VL case detection. Till date, very little effort towards development of glycan based diagnostics for PKDL case detection has been done. Consecutively, studies from our group have recently demonstrated increased titres of IgG1 containing Immune Complexes (ICs) in Indian Leishmaniasis patients with the development of a novel ELISA for serodiagnosis of both VL and PKDL [22]. With the aim to characterize novel PKDL glycan biomarkers, the present study investigates the glycosylation status of CICs of PKDL patients, followed by the development of glycan based assay for diagnosis and prognostic evaluation of Indian PKDL patients.

In the current study, we enrolled study subjects through the ongoing VL elimination programme, in endemic districts of West Bengal. Various factors like demography, ethnicity, VL history, drug and immune profile were evaluated for 90 primarily confirmed PKDL patients during the period of 2015–2016. In parallel, CICs from sera of PKDL patients along with Endemic and Non endemic controls as well as disease controls like Vitiligo and Leprosy were prepared and analyzed for development of a novel glycan based assay; the Glyco CIC assay. The assay utilizes the ability of Periodic Acid Solution (PAS) to specifically oxidize the serum CICs’ hydroxyl (-OH) groups within the carbohydrates to form aldehydes, which upon addition with Schiff reagent forms a purple color adduct, which can easily be read in a colorimeter [23]. The efficacy of this assay was further tested for its ability to distinguish PKDL positive patients from other patients with similar skin diseases like Vitiligo and Leprosy. Longitudinal monitoring of Glyco CIC titres was analyzed, to evaluate the prognostic efficiency of the assay. Further, efforts were directed towards the characterization of the novel glycan biomarkers, using SDS and gradient gel electrophoresis followed by PAS silver double staining procedures. Subsequently, Lectin blot was also performed to ascertain the glyco-profile of these newly identified proteins.

## Methods

### Study area and population

PKDL study subjects were enrolled through the ongoing VL Elimination programme (2015–2016) at block as well as sub-block levels in the state of West Bengal, India. The study subjects recruited were mainly from endemic districts of West Bengal namely ***Malda*** (25.1786°N, 88.2461°E), covering four blocks of Gazole, Bamangola, Old Malda and Habibpur, having borders with Bangladesh, ***Birbhum*** (23.8402°N, 87.6186°E) covering Golamighat and Burodanga village of Bolpur block sharing borders with Jharkhand, ***Murshidabad*** (24.2290°N, 88.2461°E) (Samserganj and Farakka blocks) sharing borders with Bangladesh. Taken together, 90PKDL cases were enrolled after taking thorough clinical examinations, information regarding history of VL andrk39 strip test positivity. After the mass survey at different localities, as per government guidelines, enrolled PKDL patients were subsequently treated at their nearest block hospitals with liposomal amphoterecin B (twice weekly at 5mg/kg body weight for three weeks)/Miltefosine (50mg/kg body weight for twelve weeks). These patients were subsequently followed up after treatment. The confirmed PKDL patients in this study belonged to different tribal communities of West Bengal. Clinico-epidemiological parameters like age, sex, medication history, were carefully recorded at individual levels. Additionally, 19 Endemic Controls(EC) subjects, 34 Non Endemic Controls (NEC) subjects were also enrolled. Further, Disease Controls included subjects with similar disease manifestations like Vitiligo (n = 46) and Leprosy (n = 37), were enrolled from outdoor/indoor departments of Dermatology and Tropical Medicine, School of Tropical Medicine, Kolkata.

### Ethics statement

Patients’ blood samples were collected after explaining the benefit and objective of the study to the patient and his/her family members by the doctors from School of Tropical Medicine, Kolkata. It was difficult to obtain written informed consents from all suspected participants who consented to take part in this study, in those cases verbal consents were obtained for rk39 RDT testing and documented through research notes. However, written informed consents were taken from all of the confirmed PKDL patients (from parents/legal guardian in case of child patients) and were assured that their identities will be kept confidential. Further, they could withdraw their names from the study at any time they wish.

After proper explanation, the volunteered participants were selected for the study. Upon agreement, the medical team members collected demographic data like sex, age, mode and duration of treatment, past history of VL/PKDL. Primary screening was done based on lesionsover trunk, peripheral parts like limbs and face (macular, nodular or polymorphic). After primary screening, 2ml of venous blood was collected in heparinized vials. Further, rK39 test was performed by trained field assistants. The ethical considerations at the study sites and outdoor and Indoor Departments of Dermatology and Tropical Medicine, School of Tropical Medicine were reviewed and approved by the Clinical Research Ethics Committee of School of Tropical Medicine, Kolkata(CREC-STM).

### Diagnosis

Suspected PKDL patients based on dermal lesions (macular, nodular or polymorphic) were carefully examined, individually by the clinicians. Venous blood (2ml) was collected in heparinized vials from consented patients. rK39 strip test (Kala Azar ^TM^ Rapid Test InBios International, Seattle, WA, USA) was performed by trained field assistants according to the standardized protocol. Confirmed PKDL patients were identified based on rK39 positivity. Continuous cold chain was maintained to deliver the blood samples at School of Tropical Medicine, Kolkata, where further experiments were performed.

### Soluble *Leishmania donovani* antigen (LDA) preparation

Briefly, crude *L*. *donovani* antigen was prepared from the *Leishmania donovani* strain MHOM/IN/83/AG83 after harvesting at the log-phase of growth. Promastigotes (1x10^7^cells/ml) were harvested in PBS (0.02M) and the pellets were resuspended in lysis buffer (20 mMTris–HCl, 40mM NaCl, pH 7.4) containing 2mM PMSF, 1mg/ml leupetin, 5mM EDTA and 5mM iodoacetamide. Further, the lysates were cryopreserved [24].

### Parasite ELISA

To determine theanti-leishmanial antibody titres (IgM, IgG and IgG subclasses), parasite ELISA was performed [25]. Precisely, LDA in phosphate buffer (0.02 M, pH 7.2) were used as coating antigen at optimal concentration (1μg/100μl/well). After blocking nonspecific sites, patient sera (diluted 1:500; 100μL/well) were added and incubated for 2hrs at 4°C. The wells were then incubated for 1hr at RT with HRP-conjugated anti-human monoclonal IgG (1:15,000) (Sigma-Aldrich, Cat**#**:A0170), monoclonal IgG1 (1:1000) (Thermo Fisher Scientific, Cat#: A-10648, USA), monoclonal IgG2 (1:1000) (Thermo Fisher Scientific, Cat#:MH1772, USA), monoclonal IgG3 (1:1000) (Thermo Fisher Scientific, Cat#:05–3620,USA), monoclonal IgG4 (1:1000) (Thermo Fisher Scientific, Cat#:A-10654,USA), and polyclonal IgM (1:10,000) (Sigma-Aldrich, Cat#:A6907), respectively, to measure the levels of IgM, IgG and IgG subclasses of anti-leishmanial antibodies with Tetramethylbenzidine (TMB). Optical density (OD) was measured at 450 nm on a micro plate reader (Erba Lisa Scan II, Germany).

### Glyco CIC assay

CICs were prepared based on protocols described by Raja *et al*, 2006 [26]. Briefly, for measuring the levels of glycosylated CICs, initially CICs were prepared with patient serum sample (10μl) diluted in Borate buffered saline (BBS) at 1:10 ratio, which was mixed with 12% PEG 6000 (Polyethylene glycol) solution in 1:1 ratio and incubated at 4°C for 45 minutes. This mixture was further centrifuged at 10,000g for 10 minutes, at room temperature (RT) to isolate the CICs. Further, acid dissociation of antigen and antibody present in CICs was performed, using glycine-HCl buffer as described by Gupta and Tan, 1981 with slight modification [27]. Concisely, the isolated CICs was dissolved in 200μl glycine-HCl, pH 1 and incubated at 47°C for 30 minutes, to dissociate the antibody-antigen complex. Consequently, the acid dissociated CICs were neutralized with 2M NaOH. Quantification of glycosylated CICs was performed according to protocol described by Schömig *et al*, 2016 [28]. Briefly, acid dissociated CICs were treated with 100μl of acidified 0.06% PAS (HIMEDIA, India) in 7% acetic acid solution (SRL, India) and incubation of the reaction mixture was performed in dark at 37°C for 90 minutes. Consequently, 100μl of Schiff reagent (HIMEDIA, India) was added to the above mixture and incubated for another 40 minutes in dark at RT, for color development. Absorbance reading was measured in colorimeter (Systronics, India*)* at 550nm and further validated on SmartSpec^TM^ Plus spectrophotometer platform (Bio-Rad, USA*)*. Mucin from porcine stomach (PSM) (Sigma-Aldrich) was used as standard in the range of 0.125 mg mL^-1^ to 1 mg mL^-1^.

### Characterization of patient CICs by SDS PAGE analysis and gradient gel electrophoresis

CICs were isolated from sera of PKDL patients by PEG precipitation and analyzed using SDS PAGE (Laemmli, 1970). Next, the gels were stained with silver staining according to the method of Merril and Harrington (1984). Further, gels were subjected to a combination of PAS silver double staining, as described by Moller *et al*, 1995 [29], to identify PKDL disease specific glycoproteins. Briefly, after electrophoresis, gels were fixed with 10% (v/v) Trichloroacetic acid (TCA) in ddH_2_O and subsequently oxidized with PAS for 40–45 minutes at RT. Further, gel was treated with acetic acid solution (1% in ddH_2_O) followed by subsequent treatment with Schiff fuchsin and Sodium metabisulphite reduction, till the bands turn magenta. Silver staining was done by subsequent wash of the PAS stained gel with acetic acid, water and dichromate fixation followed by silver nitrate treatment to develop the gel with sodium carbonate in formaldehyde solution for 40–45 minutes. The combined PAS silver staining technique enabled the visualization of intensified disease specific glycoprotein bands [29–30].

In addition, protein bands were resolved in denaturing gradient gel electrophoresis. Briefly, 5%–15% of SDS PAGE gradient gel was prepared and 50μg of CICs sample were loaded for electrophoresis, which was performed at a fixed voltage of 110V for 1.5 hrs. Protein bands in the gel were visualized by coomassie brilliant blue stain [31].

### Affinity purification of CIC antigens

To further characterize the CIC antigens, affinity purification of CIC antigens was performed using acid dissociated CICs as described by Gupta and Tan [27] with slight modification. Briefly, PKDL serum samples were pooled down (n = 20), precipitated with PEG and further treated with glycine-HCl as described earlier. CICs were affinity purified using equilibrated Protein A Sepharose 4B column (Invitrogen, USA) at RT, for 45 minutes. The antigen fraction free from antibody was thus collected. The CIC antigen fraction was subsequently dialyzed using Dialysis tubing (Sigma-Aldrich) with three PBS changes of 400 ml for 24 hours at 4°C.

Immunoglobulin fraction bound to Protein A was further incubated for another 90 minutes followed by washing in PBS (0.1 M, pH 7.2). Subsequently, the immunoglobulin was eluted with citrate buffer (0.1M, pH 3.0). Protein concentrations were measured by Lowry method [32].

### Deglycosylation of affinity purified CIC antigens

To characterize and reconfirm the presence of sugars in the affinity purified CIC antigens of PKDL patients, samples were deglycosylated using *Arthrobacter ureafaciens* neuraminidase (Sigma-Aldrich) treatment according to manufacturer’s instruction. Briefly, 50μg of affinity purified CIC antigens were treated with 2 sigma units of neuraminidase in reaction buffer and incubated at 37°C for 3 hours. The reaction mixture was heated at 100°C for 5 minutes, followed by treatment with 5 units of N-Glycosidase F (Roche, USA) in reaction buffer [20mM sodium phosphate, 10mM EDTA, 0.5% (w/v) CHAPS and 0.05% (w/v) SDS] and incubated for 18 hours at 37°C. Reaction was ultimately stopped by heating at 100°C for 5 minutes [33].

### Lectin blot of deglycosylated affinity purified CIC antigens

Glycans present in affinity purified CIC antigens were analyzed by DIG Glycan differentiation kit (Roche, USA) according to manufacturer’s instruction. Briefly, 15μg of affinity purified CIC antigens (both enzyme treated and untreated) samples were run on 7.5% SDS PAGE gel, transferred to PVDF membrane followed by Lectin blot using DIG Glycan differentiation kit. Membrane was blot dried and analyzed [34].

### Immunoblot of affinity purified CIC antigens resolved by SDS PAGE

Affinity purified CIC antigens (15μg) was electrophoresed in SDS PAGE as described earlier. Samples were run on 7.5% resolving gel prepared by 30% acrylamide and bisacrylamide solution, 1.5M Tris-HCl (pH-8.8), 10% SDS, 10% APS, and 5μl TEMED. Electrophoresis was performed on mini protean tetra cell (Bio-Rad, USA) using electrophoresis buffer (Tris 0.025M, glycine 0.19M and SDS 0.1%). Western blot was performed using affinity purified polyclonal anti-leishmanial antibody (antibodies isolated from PKDL patients) followed by treatment with HRP tagged anti-human secondary antibody monoclonal IgG (1:1000), (Sigma-Aldrich, Cat**#**:A0170) and developed with DAB substrate kit (Pierce, USA) according to manufacturer’s instruction [35].

### Statistics

Demographic analysis of the study population was performed using two tailed Chi-Square test. Statistical analysis on ELISA of IgG, IgM and IgG subclasses (IgG1, IgG2, IgG3 and IgG4), PEG index, PEG CIC and Glyco CIC assay data (2-tailed unpaired t-test for unpaired samples, with 95% confidence interval and One way ANOVA), ROC curve and longitudinal analysis (paired t-test for paired samples) was performed using Graph Pad Prism version 6.01for Windows (Graph Pad Software, San Diego, California, USA).Diagnostic performances of the assays were evaluated using MedCalc online calculator. The sensitivity and specificity for each assay were evaluated by using the equations: sensitivity = True positive cases/ (True positive cases+False negative cases)× 100% and specificity = True negative cases/ (True negative cases+ False positive cases)× 100%.Positive predictive values (PPV) and Negative predictive values (NPV) were determined using the equations: PPV = True positive cases/(True positive cases+ False positive cases) × 100% and NPV = True negative cases/(True negative cases+ False negative cases) × 100%.Receiver Operating Characteristic (ROC) curve have been constructed, summarizing the sensitivities and specificities of each assay.

## Results

### Study population

In the present study, a total of 90 PKDL cases were recruited among which,56.7% (n = 51) were male and 43.3% (n = 39) were female patients. Further, among these 90 PKDL cases, the overall inclusive median age was 20 years (range = 5–60 years). The median age of females (20 years; range = 2–56 years) was comparable to that of males (20 years; range = 5–60 years). Additionally, eighteen PKDL cases were longitudinally monitored, after administration of Liposomal Amphotericin B/Miltefosine.

Among the 90 PKDL fresh cases, majority of them had Macular forms of lesions (64.4%, n = 58) followed by Mixed/Polymorphic lesions (31.1%, n = 28), followed by Papular lesions (3.4%, n = 3) and Nodular lesions (1.1%, n = 1). Macular lesions were present in both gender (50% males, n = 29; 50% females, n *=* 29) followed by Polymorphic/Mixed forms (67.9% males, n = 19; 32.1% females, n = 9) followed by Papular forms (66.6% males, n = 2; 33.4% females, n = 1) and Nodular forms (100% male, n = 1). In the present study, 98.9% (n = 89) of PKDL patients reported history of VL. Further, among the PKDL patients with previous history of VL, 64% (n = 57) had Macular lesions, 31.5% (n = 28) had Mixed/Polymorphic lesions, 3.4% (n = 3) had Papular lesions and 1.1% (n = 1) had Nodular (Table 1).

**Table 1:** Demography details of the study population.

Subject	No. of subjects	Age (Year [Mean ± SD])	Median Age in years (Range)	Cases having history of VL	Clinically or rk39 confirmed cases
**PKDL patients**	90	23.52±11.94	20 (5–60)	89/90	88/90
**Polymorphic**	28	25.89±11.99	24 (10–60)	28/28	28/28
**Nodular**	1	40.0±0.00	Not Applicable (NA)	1/1	1/1
**Papular**	3	19.66±3.51	20 (16–23)	3/3	3/3
**Macular**	58	22.29±12.01	20 (5–56)	57/58	56/58
**Leprosy Patients**	37	32.89±18.15	28 (5–85)	0/37	0/37
**Vitiligo Patients**	46	28.60±14.64	25 (5–62)	0/46	0/46
**NEC**	34	26.67±7.62	25 (10–56)	0/34	0/34
**EC**	19	29.63±14.97	35 (4–60)	0/19	0/19

In the present study, the overall median time of manifestation of PKDL after VL treatment was 60 months (range = 12–300 months). PKDL lesions developed within 2 years in 1.2%, within 5 years in 15.5% and after five years or more in 83.3% of the cases, after apparent cure from VL. Among the enrolled PKDL cases, most of them were rk39 positive (97.8%, n = 88), however two cases recorded as rk39 negative, were confirmed parasitologically. Majority of cases with history of VL had been treated for VL with SSG (84.5%, n = 76), while the remaining were treated with Miltefosine (14.4%, n = 13) and Liposomal Amphotericin B (1.1%, n = 1). Significant association between occurrence of PKDL with previous SSG treatment in the district of Malda (χ2 = 11.57; p = 0.0007) was observed. Treatment with Miltefosine, during previous VL episodes, bears strong association for PKDL development in Birbhum district (χ2 = 10.39; p = 0.001). Similarly such association was observed also in Malda (χ2 = 15.82; p = 0.0001). Patients treated with SSG during previous VL history showed significant association to the development of macular lesion (χ2 = 3.87; p = 0.048). High correlation was observed among various lesional forms of PKDL patients, Macular/ Polymorphic/ Nodular/ Papular, based on endemic zones. Taken together, our study indicated that previous VL history, medication history, and endemicity had significant association for the development of PKDL.

### CIC PEG measurement

**Fig 1:**
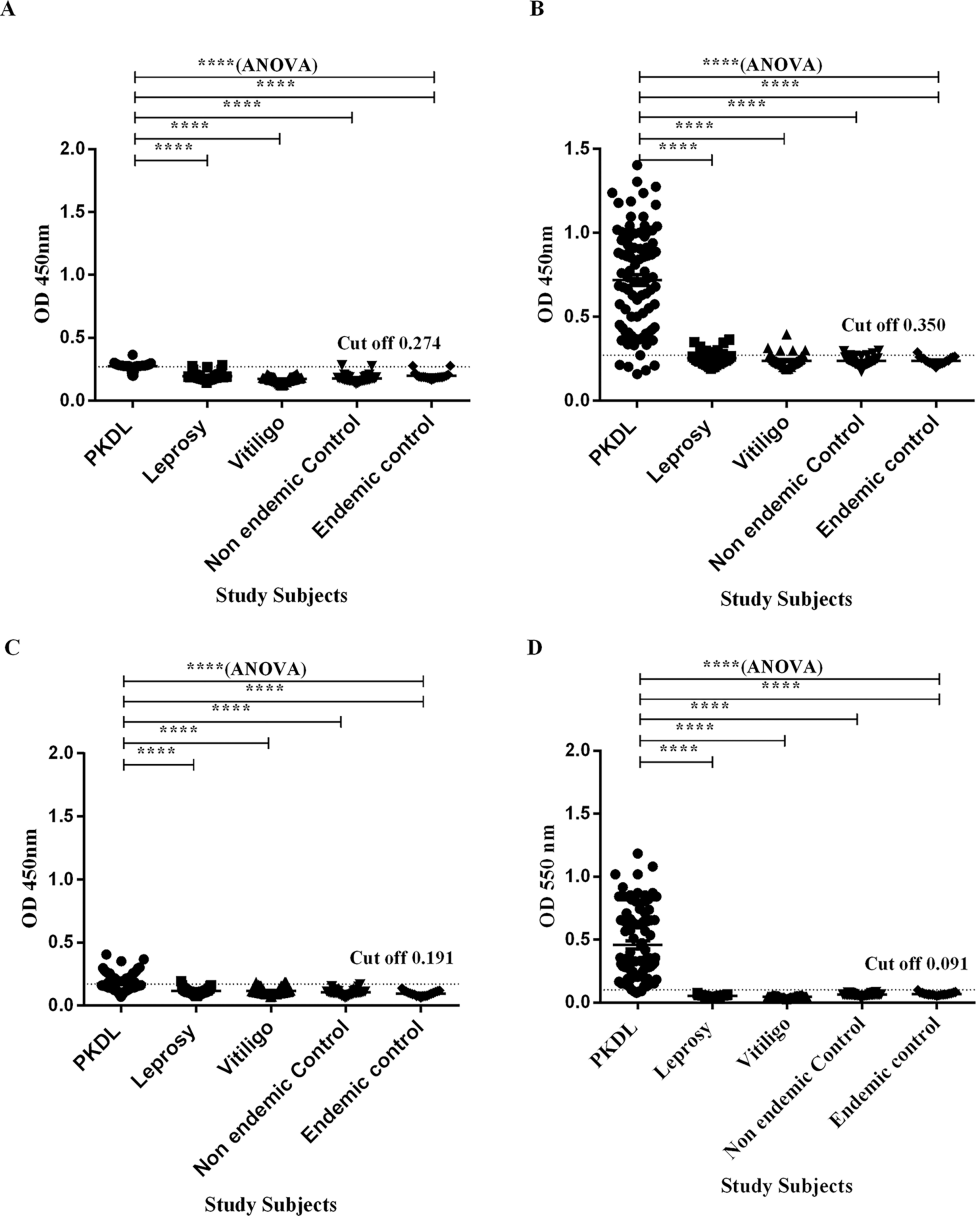
Comparative analysis of different serodiagnostic assays from serum samples obtained from PKDL patients, Healthy controls (EC and NEC) and disease control groups (Leprosy and Vitiligo). (A) Comparative evaluation of PEG CIC at 450 nm obtained from PEG precipitation of PKDL patients’ sera; cut-off 0.274. (B) Comparative evaluation of ELISA reactivity of anti-leishmanial antibody (IgG) in serum against *Leishmania* antigen; cut-off 0.350. (C) Comparative evaluation of ELISA reactivity of anti-leishmanial antibody (IgM) in serum against *Leishmania* antigen; cut-off 0.191. (D) Comparative evaluation of Glyco CIC assay based on CICs isolated from sera of PKDL patients; cut-off 0.091. The study population in all cases comprised of panel of PKDL patients (PKDL; n = 90), Endemic Healthy controls (n = 19), Non-Endemic Healthy controls (n = 34) and other disease (OD; n = 83) including Leprosy (n = 37) and Vitiligo (n = 46). Each sample was tested in triplicates and mean was taken. Each dot represent mean of single sample and the black dotted horizontal lines represent cut-off values. The cut-off value of the assays was established as means + 3 SD of 136 controls. Significance was determined by unpaired Student's t-test at 95% confidence intervals and p-values <0.05 indicate statistically significant differences.

**Fig 2:**
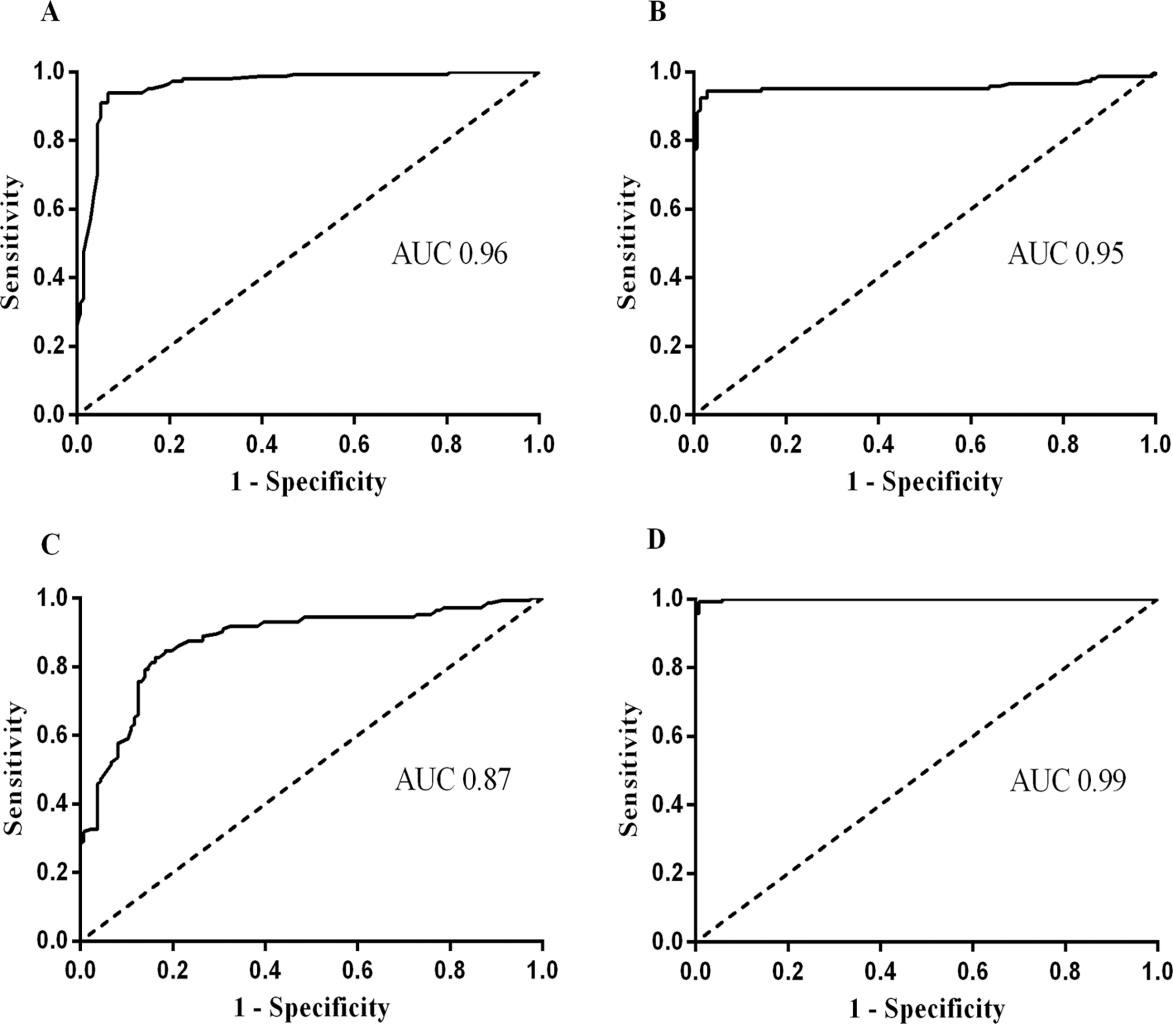
Receiver Operating Characteristics (ROC) curve for different serodiagnostic assay. (A) ROC curve obtained from PEG CIC at 450 nm values obtained from PEG precipitation of PKDL patient serum samples; Cut-off value (0.274), sensitivity (81.1%), specificity (97.1%) and AUC (0.96) were calculated for PEG CIC. (B) ROC curve obtained from the ELISA values for the detection of anti-leishmanial IgG antibody in the serum samples; Cut-off value (0.350), sensitivity (91.1%), specificity (98.5%) and AUC (0.95) were calculated for ELISA (anti-leishmanial IgG). (C) ROC curve obtained from the ELISA values for the detection of anti-leishmanial IgM antibody in the serum samples; Cut-off value (0.191), sensitivity (28.9%), specificity (99.3%) and AUC (0.87) were calculated for ELISA (anti-leishmanial IgM). (D) ROC curve obtained from the Glyco CIC assay values for the detection of glycated CICs from the serum samples; Cut-off value (0.091), sensitivity (95.6%), specificity (99.3%) and AUC (0.99) were calculated for Glyco CIC assay; All data were determined by this curve using GraphPad Prism software (version 6.01).

The PEG precipitated CIC titre values were evaluated and expressed in terms of OD at 450 nm. The ‘mean ± S.E.M’ of PEG precipitated CICs of PKDL patients was 0.276 ± 0.002 (95% CI: 0.271–0.28) as compared to controls, which were 0.197 ±0.004 (95% CI: 0.187–0.207) for Leprosy patients, 0.172 ±0.003 (95% CI: 0.165–0.178) for Vitiligo patients, 0.198 ±0.006 (95% CI: 0.184–0.213) for EC and 0.179 ±0.005 (95% CI: 0.168–0.189) for NEC (Fig 1A). The cut-off was determined as 0.274. There is an overall sensitivity of 81.1% and specificity of 97.1%. The area under the curve (AUC) is used to evaluate the accuracies of different diagnostic tests. ROC curve for PEG CIC shows AUC value of 0.96 (Fig 2A). However, there are 17 false negative cases and 4 false positive cases out of 90 cases, which could not be detected by PEG CIC test. Further, the PEG Index was estimated by modified method by Creighton *et al* [36]. The ‘mean ± S.E.M’ in terms of PEG Index of PKDL patients was 245.6 ± 2.92 (95% CI: 239.8–251.4) as compared to controls, which were 146.6 ±6.14 (95% CI: 134.2–159.1) for patients with Leprosy, 115.3 ±4.07 (95% CI: 107.1–123.5) for patients with Vitiligo,148.6 ±8.62 (95% CI: 130.4–166.7) for EC and 123.8 ±6.68 (95% CI: 110.1–137.4) for NEC.

### Comparison of anti-leishmanial antibody titres between PKDL patients and controls

*Leishmania* infection mounts humoral responses against *Leishmania* antigen in the form of immunoglobulins of isotypes IgG, IgM and IgG subclasses, which are important serological markers in the host. To assess the status of primary immunoglobulin responses, present study investigated IgG, IgM and IgG subclass antibody titres in the sera of PKDL patients with respect to EC, non PKDL disease controls like Leprosy and Vitiligo as well asNEC. Parameters involving sera dilutions and antigen concentrations were standardized to set optimal ELISA condition for the serological assay.

Anti-leishmanial antibody titres of IgG, IgG1, IgG2, IgG3 and IgG4 in sera of PKDL patients showed enhanced responses. Reactivity of *Leishmania* antigen was also investigated against antibody Isotype of IgM present in the sera samples. The performances of the sensitivities and the specificities of the ELISA with sera from patients with PKDL were also evaluated. The reactivity of the IgG, IgM, and IgG subclass antibodies from the sera of patients with PKDL (n = 90) were significantly higher than those of the sera from the controls, including NEC, EC, and patients with Leprosy and other skin disease like Vitiligo (p<0.0001). The ‘mean ± S.E.M’ of anti- leishmanial IgG antibody titres in sera of PKDL patients were 0.718 ± 0.032 (95% CI: 0.65–0.78) in comparison to the controls, which were 0.25 ± 0.007 (95% CI: 0.23–0.26) for patients with Leprosy, 0.23 ± 0.005 (95% CI: 0.22–0.24) for patients with Vitiligo, 0.23 ± 0.004 (95% CI: 0.22–0.24) for EC and 0.23 ± 0.004 (95% CI: 0.22–0.24) for NEC (Fig 1B). There is a sensitivity and specificity of 91.1% and 98.5%, respectively for IgG ELISA. Thus, among the 90 PKDL cases, 8 false negative cases were reported by IgG ELISA assay with 2 false positive cases among control cases. The cut-off was determined as 0.350. Through the assessment of ROC curve, AUC value of 0.95 was obtained, showing good performance of IgG ELISA for discriminating PKDL cases from controls (Fig 2B).

To differentiate PKDL serologically from the control populations, the *Leishmania* antigen specific isotype IgG titres were analyzed. High levels of anti-leishmanial IgG antibodies were observed in the sera of PKDL patients with respect to EC (p<0.0001) as well as disease controls (p<0.0001).

The *Leishmania* antigen specific IgG1 and IgG3 responses of PKDL patients showed moderately elevated antibody titres with respect to both controls (Table 2).

**Table 2:** Distribution of IgG subclass anti-leishmanial antibody titres of the study population.

Subject	IgG1 (OD_450nm_ [Mean ± S.E.M])	IgG2 (OD_450nm_ [Mean ± S.E.M])	IgG3 (OD_450nm_ [Mean ± S.E.M])	IgG4 (OD_450nm_ [Mean ± S.E.M])
**PKDL patients**	0.527 ± 0.020	0.355 ± 0.010	0.474 ± 0.030	0.201 ± 0.005
**Leprosy Patients**	0.340 ± 0.024	0.306 ± 0.012	0.266 ± 0.031	0.199± 0.007
**Vitiligo Patients**	0.320 ± 0.028	0.276 ± 0.023	0.332± 0.033	0.186 ± 0.011
**Healthy Controls**	0.335 ± 0.034	0.242 ± 0.011	0.311 ± 0.037	0.203 ± 0.014
**Healthy Endemic controls**	0.346 ± 0.016	0.275 ± 0.020	0.325 ± 0.017	0.189 ± 0.008

Moreover, there was moderately significant increase in anti-leishmanial IgM antibody titre levels, which was observed in sera of patients with PKDL (0.175 ± 0.006) (95%CI:0.16–0.18) patients as compared to control groups, including patients with Leprosy (0.115 ± 0.003) (95%CI:0.10–0.12),patients with Vitiligo (0.116±0.004) (95% CI: 0.10–0.12), EC(0.095 ± 0.004) (95% CI: 0.08–0.10) and NEC (0.105 ± 0.003) (95%CI:0.09–0.11) (Fig 1C). The cut-off was determined as 0.191 with an assay sensitivity of 28.9% and specificity of 99.3%. IgM ELISA demonstrated 64 false negative cases and 1 false positive case among 90PKDL cases and 136 controls. Further, ROC curve for IgM ELISA showed AUC value of 0.87 (Fig 2C), suggesting poor performance than IgG ELISA.

### Glyco CIC assay

To quantify the glycosylated CICs in PEG precipitated sera, the Glyco CIC assay was employed. The assay was performed using only 10 μl of sera. The measurement was taken at 550 nm wavelength. Parameters such as hours of PAS and Schiff reagent treatment were optimized to set standardized assay time for the rapidity and reproducibility of the diagnosis. Finally, all sera samples were separated, from finger pricked amount of whole blood, for examination using Glyco CIC assay. The ‘mean ± S.E.M’ of Glyco CIC assay of PKDL patients was 0.458± 0.03 (95% CI: 0.39–0.51) as compared to controls, which was 0.051± 0.001 (95% CI: 0.04–0.05) for patients with Leprosy, 0.046± 0.001 (95% CI: 0.044–0.048) for patients with Vitiligo, 0.069± 0.002 (95% CI: 0.06–0.07) for EC and 0.062± 0.001 (95% CI: 0.05–0.06) for NEC (Fig 1D). The test cut-off point was determined as 0.091. Sera from PKDL patients demonstrated significantly stronger (p<0.0001) recognition of glycosylated CICs than any of the control groups. Thus, complete agreement (κ = 0.944) was observed between the Glyco CIC assay and the reference rk39 test for diagnosis of PKDL. The overall sensitivity of the assay was 95.6% and specificity was 99.3%. There were only 4 false negatives and 1 false positive from 90 PKDL cases. ROC curve for Glyco CIC assay shows AUC value of 0.99, indicating excellent diagnostic efficacies (Fig 2D). Additionally, two PKDL cases which were negative to rK39 tests, but were confirmed parasitologically, were also found positive through Glyco CIC assay.

### Glyco characterization of CIC

**Fig 3:**
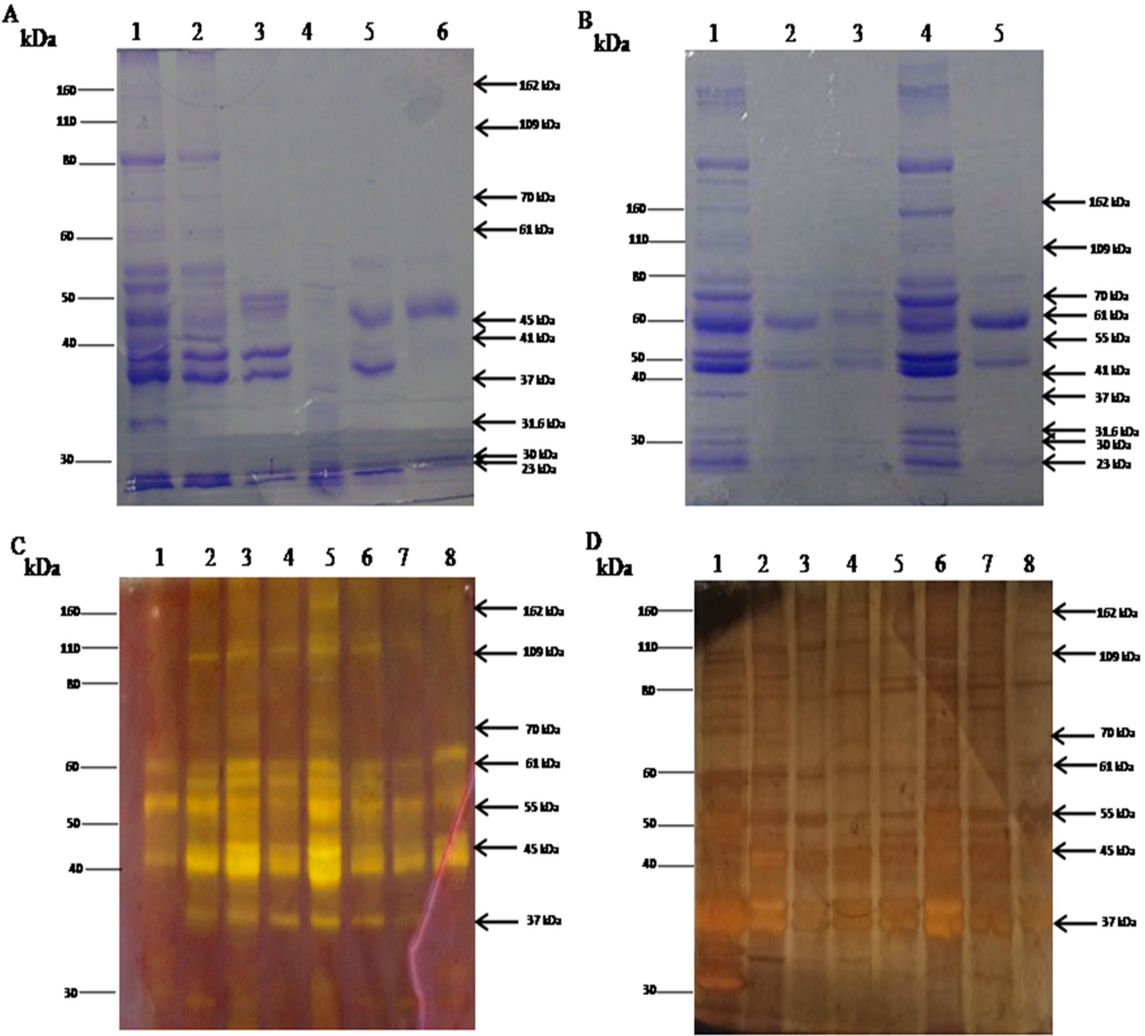
Circulating immune complexes (CICs) status in PKDL positive cases compared to other similar skin diseases. (A) CICs profile in different forms of Leishmaniasis. Lane 1 (Malda) represents PKDL patient profile; Lane 2 represents VL patient (Murshidabad); Lane 3 represents Cutaneous Leishmaniasis (CL) patient; Lane 4 represents *Leishmania donovani* antigen; Lane 5 represents Leprosy and Lane 6 represents Vitiligo patient. (B) CICs profile in Gradient gel electrophoresis (5%–15%). Lane 1 (Malda) and Lane 4 (Birbhum) represents untreated PKDL; Lane 2 (Malda), Lane 3 (Malda) and Lane 5 (Malda) represents treated PKDL patient; Black arrows mark the protein bands of 162, 109, 70, 61, 45, 41, 37, 31.6, 30, and 23 kDa, which is present in all PKDL patients (untreated) (C) CICs profile of sera from patients with different diseases run on a 7.5% Silver stained SDS PAGE. Lane 1 represents VL patient; Lane 2 and 3 represents Vitiligo patient; Lane 4 and 5 represents Leprosy patient; Lane 6 (Malda) represents polymorphic PKDL patient; Lane 7 (Murshidabad) represents macular PKDL patient and Lane 8 represents Non Endemic control. Corresponding protein bands are absent in Leprosy, Vitiligo and Non Endemic control. (D) CICs in sera of different forms of PKDL run on a 7.5% PAS-Silver double stained SDS PAGE. Lane 1 represents Healthy control; Lane 2 (Birbhum), Lane 3 (Birbhum), Lane 4 (Malda) and Lane 6 (Malda) represents macular PKDL patients; Lane 5 (Murshidabad) and Lane 7 (Malda) represents polymorphic PKDL patient; Lane 8 represents Leprosy patient; Black arrows mark the protein bands of 162, 109, 70, 61, 55, 45, and 37 kDa, that are common to all PKDL patients.

To characterize the CICs among different forms of Leishmaniasis as compared to other cross diseases and healthy controls, initially, PEG precipitated CICs from sera of PKDL, VL and Cutaneous Leishmaniasis (CL) patients, cross disease control (Leprosy, Vitiligo) and NEC were analyzed using one dimensional SDS PAGE, which revealed a differential banding pattern among different types of disease manifestations (Fig 3A). Gradient SDS gel electrophoresis of PKDL PEG precipitated CICs indicated the exclusive presence of ten bands of molecular weight (MW) 162, 109, 70, 61, 45, 41, 37, 31.6, 30, 23 kDa (Fig 3B Lane 1,4). Importantly, these bands were either absent or present with decreased intensity in CICs of treated PKDL patients (Fig 3B Lane 2, 3, 5 respectively). Also, CICs of VL patients and *Leishmania* membrane protein (*L*. *donovani* strain Ag83) specifically indicated the presence of 109 kDa band, which was also present in CICs of PKDL patients (Fig 3A Lane 2, 4 respectively). When SDS gel were subjected to silver staining alone, CICs from PKDL patients indicated presence of extra band of molecular weight (MW) 55 kDa along with 162, 109, 70, 61, 45, 41, 37, 31.6, 30, 23 kDa bands (Fig 3C Lane 6,7). For further characterization of specific protein bands in CICs of PKDL patients, gels were further subjected to PAS silver double staining, which revealed seven Glycoprotein bands of MW 162, 109, 70, 61, 55, 45 and 37 kDa (Fig 3D Lane 2–7). CICs of disease control and NEC indicated total absence of the corresponding bands (Fig 3D Lane 1, 8).

**Fig 4:**
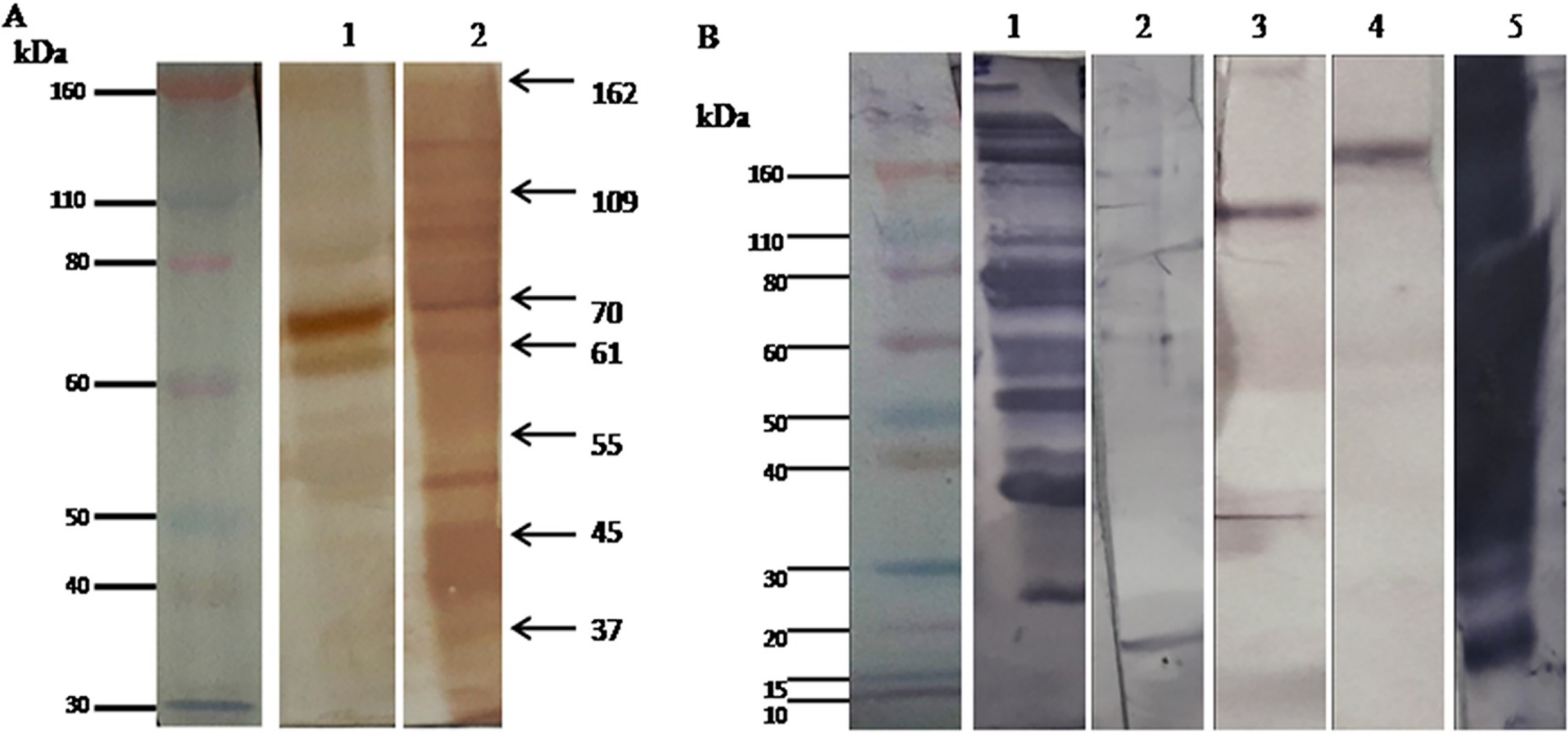
Glycation status of circulating immune complexes (CICs) among pooled PKDL positive cases. (A) Immunoblot from CICs of PKDL patients with purified anti-leishmanial antibody. Lane 1 represents purified CICs (pooled down; n = 20) of PKDL patients; Lane 2 represents *L*. *donovani* antigen; Black arrows mark the bands of MW 162, 109, 70, 61, 55, 45 and 37 kDa that are common among both PKDL patients and *L*. *donovani* antigen. (B) Lectin blot was done from purified CICs (pooled down; n = 20) of PKDL patients with DIG Glycan differentiation kit. Lane 1 represents CICs blotted with lectin SNA; Lane 2 represents enzyme (neuraminidase and N- Glycosidase F) treated purified CICs (pooled down; n = 20) of PKDL patients; Lane 3 represents CICs blotted with lectin GNA; Lane 4 represents CICs blotted with lectin DSA; Lane 5 represents Fetuin.

To characterize the antigens, PEG precipitated CICs from 20 PKDL patients were pooled down, acid dissociated and affinity purified using Protein A Sepharose 4B. The immunogenic nature of the affinity purified CIC antigens was deciphered through immunoblot analysis using purified anti-leishmanial antibody (Fig 4A). Protein bands of molecular weight 162, 109, 70, 61, 55, 45 and 37 kDa (Fig 4A Lane 1) in the affinity purified CIC antigens corresponded to similar molecular weight protein bands present in *L*. *donovani* antigen (Fig 4A Lane 2).

To further validate the presence of glycosylation in CIC antigens, lectin blot was performed (Fig 4B Lane 1, Lane 3, Lane 4) with lectin SNA (binding specificity to SAα2–6 Gal), GNA (binding specificity to Man) and DSA (binding specificity to Gal1-4GlcNAc) respectively, using the DIG Glycan differentiation kit, which revealed the presence of several protein bands of molecular weight (MW) ranging from 20–260 kDa (Fig 4B Lane 1). Additionally, to reconfirm the glycan nature of the CIC antigens, CICs were deglycosylated, using enzyme Neuraminidase and N Glycosidase F (Fig 4B Lane 2), which revealed the complete disappearance of all the identified bands, confirming the glycan nature of the identified affinity purified CIC antigens. Fetuin (Fig 4B Lane 5) was used as positive control.

### Prognostic potential of Glyco CIC assay vs. conventional parasite ELISA

**Fig 5:**
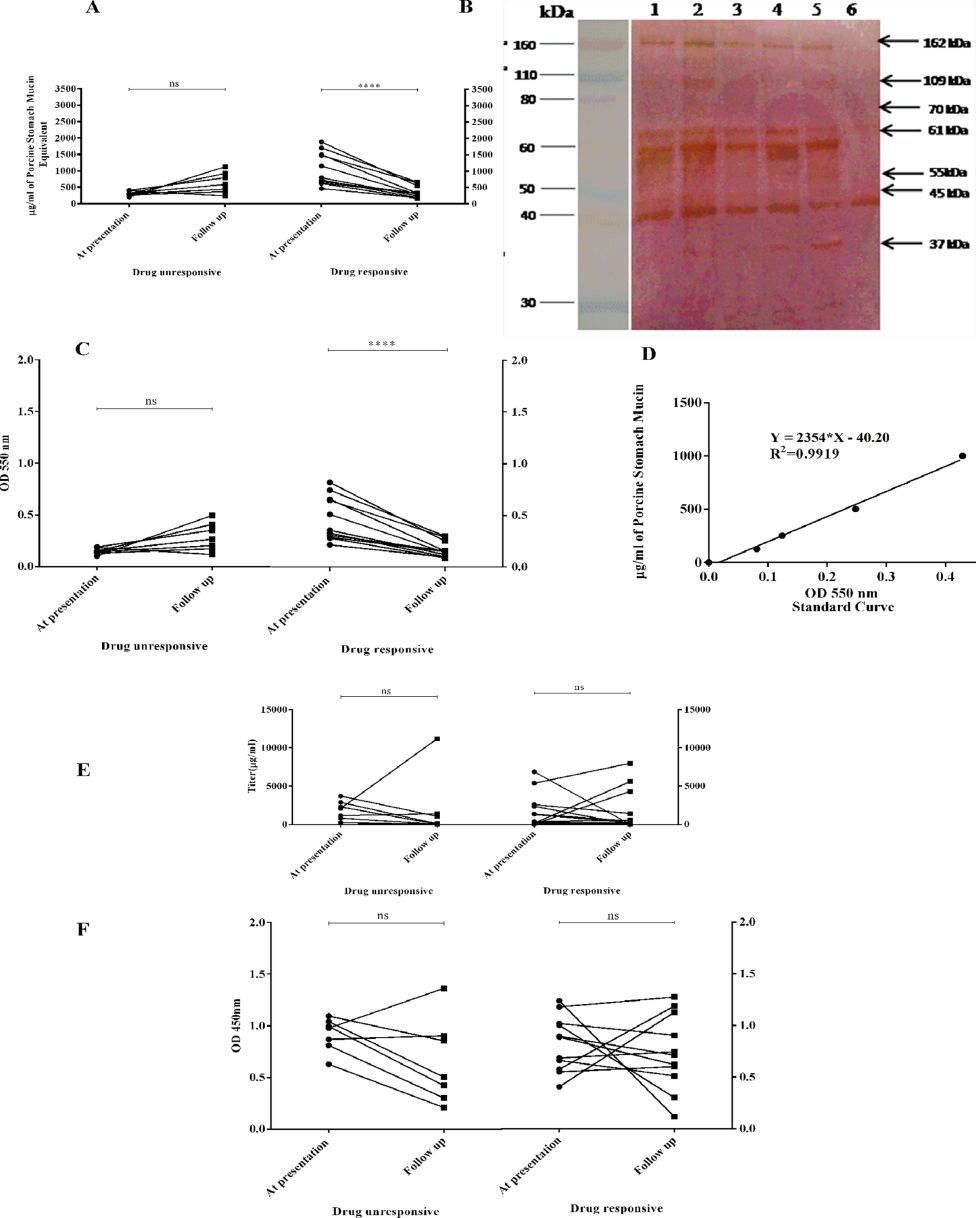
Comparison of Glyco CIC assay and parasite ELISA: Longitudinal monitoring of PKDL patients. (A) Mucin from Porcine Stomach (PSM) equivalent value as obtained through Glyco CIC assay for drug unresponsive and responsive PKDL patients; at presentation and follow up. (B) CICs profile of PKDL patients before and after treatment. Lane 1 (Malda), Lane 3 (Malda) and Lane 5 (Malda) represents PKDL patients (before treatment); Lane 2 (Malda), Lane 4 (Malda) and Lane 6 (Malda) represents PKDL patients (after treatment); Black arrows mark the protein bands of 162, 109, 70, 61, 55, 45, and 37 kDa that are common to PKDL patients. (C) Absorbance value ofGlyco CIC assay for drug unresponsive and responsive PKDL patients; at presentation and follow up at OD 550 nm. (D) Standard curve for PSM (μg/ml). (E) *Leishmania* Antigen-specific serum IgG antibody titres and (F) Corresponding absorbance values of Anti Leishmanial IgG in drug unresponsive and responsive PKDL patients; at presentation and follow up.

Evaluation of Glyco CIC assay and anti-leishmanial antibody ELISA was performed to determine their prognostic potential among 18 paired PKDL samples at day 0 and *>*180 after drug administration. The Glyco CIC assay showed that all the 11 patients after treatment with Miltefosine (drug responsive group) demonstrated 3 fold decrease in the values (1062 ± 151.7 vs. 354 ± 54.7 μg/μl PSM equivalent; p<0.0001), indicating that these patients were responding to treatment, which was also confirmed clinically. Further, the Glyco CIC assay could also successfully identify the 7 drug unresponsive patients (receiving LAMB) thus, indicating its utility for therapeutic monitoring (Fig 5A and 5C). Whereas, the conventional anti-leishmanial antibody based ELISA failed to differentiate follow-up from baseline cases in both drug responsive and unresponsive groups, where the mean titre showed insignificant differences for both drug unresponsive (p = 0.95) and drug responsive (p = 0.99) cases (Fig 5E and 5F).

## Discussion

One of the earliest records of Indian PKDL cases acting as the sole reservoir for transmission of the fatal parasitic disease VL, was made by Addy & Nandy in 1992 [37]. They demonstrated the outbreak of the disease in two foci, one of these foci was in Malda district, in northern West Bengal and the other was located in the south of West Bengal. This study highlighted the important role played by PKDL cases in anthroponotic transmission of VL in West Bengal, India. Since then, there have been very few reports on PKDL epidemiology or surveillance from this part of India, until recently however, Ganguly *et al* 2015 [38] reported a large number of PKDL cases from endemic areas of Malda district. Subsequently, Saha*et al* 2017 [39] have also reported a huge number of asymptomatic cases from these endemic areas. Thus, the accelerated VL elimination programme has resulted in increased case detection. The ongoing VL elimination survey in West Bengal has recorded a large number of cases in eleven endemic districts. Active survey in West Bengal has resulted in identification of 248 new cases in 2015 and 240 new cases in 2016 [40]. However, one of the major challenges faced by this programme is accurate and reliable diagnosis of PKDL cases, as a large percentage of the patient population have no history of VL and are often rk39 negative. The problem is further compounded with the fact that our available diagnostic arsenal fails to distinguish between fresh and cured cases. Further, the treatment of PKDL is also difficult and is yet not standardized, often the regime is prolonged and frequently patients discontinue therapy and thus, continue to serve as reservoirs of infection. Additionally, the drug responsiveness of PKDL patients also varies, where cure is early in some patients, whereas others are late responders. Thus, an appropriate tool for therapeutic monitoring is much needed, which would suffice the current need of the hour for successful implementation of the elimination programme. Government of India action plan encompassed major commitment towards elimination of VL by 2017 through memorandum of understanding signed by five countries [41]. Unfortunately, even after the active drive, India and Bangladesh still have a prevalence of around 8 to 16 cases per 10,000 populations in some of the endemic districts at block levels [42]. From the diagnostic point of view, parasite identification in slit skin smears and culture methods are confirmatory, but lack sensitivity, being only 40%-60% sensitive in case of nodular lesions of PKDL [43,44], whereas PCR demands non field set up and highly skilled personnel under stringent laboratory conditions, though being 93.8% sensitive [45]. Serological methods like rK39 dipstick tests, DAT, ELISA and Leishmanin tests although bears 91%-100% sensitivity and 0–100% in terms of differential diagnosis of Leprosy, Vitiligo and other diseases in the endemic zones, but have zonal variability of diagnostic efficacies. Serological diagnosis could only establish detection of 20% of PKDL cases, lacking reports of VL history and they seldom distinguish treated cases from early PKDL cases as these are all antibody based tests having persistence of antibody titres even after cure [46].

PKDL is a chronic disease. In India, it usually develops after several years of cure from VL. The causative parasite *i*.*e*., *Leishmania donovani* which persists in the skin comes in contact with high levels of antibody, but are not removed due to defective clearance and thus drive the development of ICs in circulation [47]. Formation of ICs is a normal part of the immune defense to remove pathogen. More recently, Jamal *et al*, 2017 [48] have demonstrated a 36 fold higher ICs in human VL subjects. In our present study, we observed almost 5 fold higher ICs in human PKDL subjects. Aberrant glycosylation of proteins in ICs often play important role in pathogenesis of various disease conditions like SLE, Rheumatoid Arthritis and Cancer [49–52]. Recently, CICs has also been identified to play active role in *Leishmania* infection [22, 53]. In Rheumatoid Arthritis, there have been implications of glycosylation alterations in ICs progression to disease manifestation. Overwhelming data supports the role of glycosylation of IgG in human diseases involving human Rheumatoid Arthritis [54–55]. Recent studies have also demonstrated that glycosylation of protein antigens can immensely affect adaptive immune responses [56,57]. Taken together, glycoproteins have been utilized as diagnostic markers in innumerable number of diseases as they are known to possess disease specificity and are very reliable [58].

Preliminary studies from our laboratory have indicated that PKDL CICs are glycosylated [22]. Based on the above strong background literature, we explored the potential of glycated CICs towards PKDL disease diagnosis and prognosis. We developed a glycan based assay which quantifies the amount of glycosylated CICs in PKDL patients and thus helps in the diagnosis of these patients. The study was conducted on PKDL subjects collected from endemic districts of West Bengal. Our data further, revealed the presence of specific glycoproteins among PKDL positive individuals with various degrees of dermal manifestations such as Macular lesions, Papular, Mixed and Polymorphic lesions including Nodular types of lesions. These findings are consistent with our earlier report of identification and characterization of CICs in patients with VL and PKDL [22]. The enrolled PKDL patients in our study were mainly from north of West Bengal covering districts like Malda. Districts like Birbhum and Murshidabad were also surveyed, though the disease burden was comparatively less there. Most of the cases registered were macular PKDL (64.4%). Diagnosis of these cases are particularly difficult as the clinical presentations bear very close resemblance to cross diseases like Vitiligo or Leprosy and have scanty parasite load. The rK39 is undoubtedly a useful test, especially in a field setting, but it has low sensitivity for Asian macular PKDL [59]. In the present study, we employed the Glyco CIC assay to identify both rK39 positive and negative PKDL samples. The assay could successfully identify two parasitologically confirmed rK39 negative cases. For evaluating the diagnostic potential of our newly developed Glyco CIC assay, we compared it with other available serodiagnostic assays, namely PEG CIC assay, conventional parasite ELISA (anti-leishmanial IgM and IgG). The Glyco CIC assay yields superior sensitivity of 95.6% and specificity of 99.3%, whereas the NPV was 97.1% and PPV was 98.9%. On the other hand PEG CIC assay yielded a mere sensitivity of only 81.1% and specificity of 97.1%. Available and mostly practiced serodiagnostic assay like ELISA which employs *Leishmania* antigen for detection of serum anti-leishmanial antibodies level yielded a sensitivity of 91.1% and specificity of 98.5% for IgG and the corresponding sensitivity and specificity for IgM were 28.9% and 99.3%, respectively (Table 3).

**Table 3:** Comparative diagnostic performances of the assays employed in the study population.

Diagnostic Tests	Sensitivity % (CI 95%)	Specificity % (CI 95%)	Positive Predictive Value % (CI 95%) (PPV)	Negative Predictive Value % (CI 95%) (NPV)
**PEG CIC Assay**	81.1 (71.5–88.6)	97.1 (92.6–99.2)	94.8 (87.4–97.9)	88.6 (83.5–92.3)
**Parasite IgM ELISA**	28.9 (19.8–39.4)	99.3 (95.9–99.9)	96.3 (78.2–99.8)	67.8 (64.9–70.7)
**Parasite IgG ELISA**	91.1 (83.2–96.0)	98.5 (94.8–99.8)	97.6 (91.1–99.4)	94.4 (89.6–97.0)
**Glyco CIC assay**	95.6 (89–98.8)	99.3 (95.9–99.9)	98.9 (92.4–99.8)	97.1 (92.8–98.9)

Thus, Glyco CIC assay had remarkable sensitivity and specificity limits, though it failed to diagnose 2 macular and 2 polymorphic PKDL cases from Malda district. Interestingly, glycated CICs as quantified by our new assay was significantly high for PKDL patients in all the studied districts namely Malda (p<0.0001), Birbhum (p<0.0001) and Murshidabad (p<0.0001). Additionally, the PKDL CICs titre OD as evaluated through Glyco CIC assay scored 5.03 fold the OD cut off value. We further statistically evaluated our findings based on ROC curve for Glyco CIC assay compared with available serodiagnostic assay and obtained AUC value of 0.99 for Glyco CIC assay, 0.96 for PEG CIC at 450 nm, 0.95 and 0.87 for ELISA based antibody titres of IgG and IgM, respectively (Fig 2). Thus, taken together, Glyco CIC assay had an impressive diagnostic efficacy as compared to the other available assays.

As our study demonstrated high titres of glycosylated CICs in PKDL patients, we then systematically characterized the glycosylation profile of PKDL CICs. SDS and gradient gel analysis revealed the presence often major protein bands of molecular weight (MW) 162 kDa, 109 kDa, 70 kDa, 61 kDa, 45 kDa, 41 kDa, 37 kDa, 31.6 kDa, 30 kDa and 23 kDa exclusively in PKDL patients. Among them, distinct seven bands were found to be glycoprotein in nature as demonstrated by PAS silver double stain and lectin blot (Fig 3D, Fig 4B). Notably, the 162 kDa, 109 kDa, 70 kDa MW CIC protein bands have similar MW as glycoproteins found on amastigotes and promastigotes of *Leishmania donovani* [60], whereas other protein bands like 61 kDa, 55 kDa, 45 kDa and 37 kDa had MW similar to those found in *Leishmania* antigen [48]. All the above mentioned seven protein bands were present in immunoblot (Fig 4A). These glycoprotein biomarkers were present in all the patients enrolled from the different districts of West Bengal. These bands were totally absent in other cross diseases (Leprosy and Vitiligo) and Healthy controls (EC and NEC). Further, longitudinal monitoring of PKDL patients revealed the disappearance (162 kDa, 109 kDa, 70 kDa, 45 kDa, 37 kDa) /decreased (61 kDa) band intensity in drug responsive patients, whereas these bands persisted in the drug unresponsive group (Fig 5B). This thus, highlighted the prognostic application of these newly identified PKDL glycoprotein biomarkers.

Taken together, our study demonstrated that the newly developed Glyco CIC assay could successfully differentiate between PKDL drug responsive vs. drug un-responsive patients. Thus, among the 18 patients, who were monitored longitudinally, eleven PKDL patients were recognized as clinically cured and had no dermal lesions after treatment with Miltefosine. These patients showed almost 3 fold reduced PSM equivalent CICs concentration (Fig 5). Whereas,7 such patients, who were kept under LAMB regime, did not show regression of the dermal lesions, and showed almost 2.03 fold increased PSM equivalent CICs concentration in their follow up, reflecting persistence of the infection (Fig 5). PSM has been used as a standard for our newly developed Glyco CIC assay, as it is a rich source of naturally occurring glycoproteins with high carbohydrate content. The standard has helped us to quantitate the amount of glycosylated CICs present in the sample. Glyco CIC assay may thus, serves as an efficient prognostic evaluator of PKDL patients. In contrast, the conventional parasite ELISA (IgG) failed to distinguish between these groups (Fig 5). The titre value ‘mean ± S.E.M’ value of 1881 ± 462.3 μg/ml was obtained for drug unresponsive cases at presentation with respect to follow up mean titre value of 1970 ± 1555 μg/ml. Similarly, for drug responsive cases ‘mean ± S.E.M’ value of 1879 ± 692.9μg/ml at presentation with respect to follow up mean titre value of 1881 ± 835.4 μg/ml was observed (Fig 5E).

Glyco CIC assay utilizes the glycation status of CICs for identification and prognostic evaluation of PKDL patients. The assay is efficient and very cheap. Although PCR assay for identification of parasite load in PKDL patient stands as gold standard, but it requires central laboratory facilities. Also, it requires high end instruments and expertise. Glyco CIC assay on the other hand, provides an easy alternative for diagnostic and prognostic evaluation of PKDL patients. It does not involve much expertise and high end instruments. Also, the assay reagents are cost effective and can be used for large sample sizes.

Our findings highlight the efficacy of Glyco CIC assay over available invasive methods like punch biopsy and tissue aspiration. The assay has potential for working both as a diagnostic and prognostic evaluator of Indian PKDL patients. It may also be used as an alternative to highly invasive tissue aspiration techniques particularly in primary health care centers. The assay requires very less blood sample, hence finds advantage over painful invasive techniques. Thus, the assay can be a potent tool for efficient case detection and could go hand in hand with the VL management and elimination program.

## Supporting information

S1 Checklist (DOC)

S1 Fig (DOCX)
