# Supplementary material for: Glycoproteins in circulating immune complexes are biomarkers of patients with Indian PKDL: A study from endemic districts of West Bengal, India
Source: PLoS One. 2018 Feb 8;13(2):e0192302. doi: 10.1371/journal.pone.0192302 (PMC5805291; doi:10.1371/journal.pone.0192302)
Supplement: S1 Checklist — (DOC) [file pone.0192302.s001.doc]

| **S1 checklist** **STARD CHECKLIST *(version 2015)*** | **Section & Topic** | **No** | **Item** | **Reported on page #** |
| --- | --- | --- | --- | --- |
|  |  |  |  |  |
|  | **TITLE OR ABSTRACT** |  |  |  |
|  |  | **1** | Identification as a study of diagnostic accuracy using at least one measure of accuracy  (such as sensitivity, specificity, predictive values, or AUC)  **The Identification of Biomarkers along with development and evaluation of Glyco CIC assay for diagnostics of Indian PKDL.** | **3-4** |
|  | **ABSTRACT** |  |  |  |
|  |  | **2** | Structured summary of study design, methods, results, and conclusions  (for specific guidance, see STARD for Abstracts)  **The article is a study of diagnostic accuracy using measures of sensitivity, specificity and AUC.**  **Provides summary of study design, results and conclusion** | **3-4** |
|  | **INTRODUCTION** |  |  |  |
|  |  | **3** | Scientific and clinical background, including the intended use and clinical role of the index test  **The background of the work such as routine diagnostics and other similar test have been explained** | **5-7** |
|  |  | **4** | Study objectives and hypotheses  **Study objectives was development of Glyco CIC assay for Indian PKDL diagnosis and its comparison with reference test rK39 RDT and additional test such as Parasite ELISA.** | **6-7** |
|  | **METHODS** |  |  |  |
|  | *Study design* | **5** | Whether data collection was planned before the index test and reference standard  were performed (prospective study) or after (retrospective study)  **This study is a prospective study which were planned before the reference and index tests were performed** | **7-8** |
|  | *Participants* | **6** | Eligibility criteria  **Confirmed PKDL patients of both genders living in and around the endemic area and ready to give consent.** | **8-9** |
|  |  | **7** | On what basis potentially eligible participants were identified  (such as symptoms, results from previous tests, inclusion in registry)  **Participants were recruited based on clinical symptoms, other test results and rK39 rapid tests. Peripheral blood samples were collected.** | **8-10** |
|  |  | **8** | Where and when potentially eligible participants were identified (setting, location and dates)  **Blood samples of confirmed cases and other diseases were collected through the ongoing VL elimination programme in the state of West Bengal where reference test were done. index test were conducted in School of Tropical Medicine.** | **8-9** |
|  |  | **9** | Whether participants formed a consecutive, random or convenience series  **Reference test was performed as a mandatory part of ongoing VL elimination programme.** | **9** |
|  | *Test methods* | **10a** | Index test, in sufficient detail to allow replication  **Glyco CIC assay were developed and evaluated for diagnosis of Indian PKDL. Details available in the manuscript.** | **10-11** |
|  |  | **10b** | Reference standard, in sufficient detail to allow replication  **Reference standard is rk39RDT for detection of antileishmanial antibodies** | **9** |
|  |  | **11** | Rationale for choosing the reference standard (if alternatives exist)  **Diagnosis of PKDL is through rk39RDT which is a mandatory part of ongoing VL elimination programme. This is the first line of diagnostics routinely used by the practitioners.** | **9** |
|  |  | **12a** | Definition of and rationale for test positivity cut-offs or result categories  of the index test, distinguishing pre-specified from exploratory  **Diagnostic accuracy of Glyco CIC assay was calculated using Graphpad prism software and it was not pre-specified as the index test is developed during the study.** | **10-11** |
|  |  | **12b** | Definition of and rationale for test positivity cut-offs or result categories  of the reference standard, distinguishing pre-specified from exploratory  **Reference test was used for screening clinically suspect population** | **9** |
|  |  | **13a** | Whether clinical information and reference standard results were available  to the performers/readers of the index test  **Yes, as index tests were performed with samples which were performed with reference test.** | **9-10** |
|  |  | **13b** | Whether clinical information and index test results were available  to the assessors of the reference standard  **Yes** | **7-9** |
|  | *Analysis* | **14** | Methods for estimating or comparing measures of diagnostic accuracy  **ROC curve were plotted and sensitivity, specificity, cut-offs and AUC of the tests were calculated along with NPV and PPV.** | **17-20,26-27** |
|  |  | **15** | How indeterminate index test or reference standard results were handled  **Indeterminate reference test were discarded from the study where as uncertain results of index tests were re-evaluated.** | **15** |
|  |  | **16** | How missing data on the index test and reference standard were handled | **NA** |
|  |  | **17** | Any analyses of variability in diagnostic accuracy, distinguishing pre-specified from exploratory  **NO** | **NA** |
|  |  | **18** | Intended sample size and how it was determined  **With the anticipation of 95% sensitivity with 95% confidence interval we successfully evaluated 90 samples.** | **7-8** |
|  | **RESULTS** |  |  |  |
|  | *Participants* | **19** | Flow of participants, using a diagram  **Presented in S1 Fig** | **S1 Fig , 38-39** |
|  |  | **20** | Baseline demographic and clinical characteristics of participants  **As detailed in the manuscript** | **15-16** |
|  |  | **21a** | Distribution of severity of disease in those with the target condition  **Dermal manifestation of PKDL as detailed in the manuscript** | **15-16** |
|  |  | **21b** | Distribution ofalternative diagnoses in those without the target condition  **Performed with Leprosy and Vitiligo.** | **7-8** |
|  |  | **22** | Time interval and any clinical interventions between index test and reference standard  **Both the tests were performed before the treatment started, reference test followed by index test within 72 hrs.** | **9** |
|  | *Test results* | **23** | Cross tabulation of the index test results (or their distribution)  by the results of the reference standard  **Results of both the index test Glyco CIC assay were compared to rK39 strip test result.** | **20-21** |
|  |  | **24** | Estimates of diagnostic accuracy and their precision (such as 95% confidence intervals)  More than 95% sensitivity and specificity were estimated for the test with 95% confidence interval . | **Fig 1, Fig 2** |
|  |  | **25** | Any adverse events from performing the index test or the reference standard  **NO** | **NA** |
|  | **DISCUSSION** |  |  |  |
|  |  | **26** | Study limitations, including sources of potential bias, statistical uncertainty, and generalisability  **Study was conducted with confirmed cases in reference standard test. Therefore reference test was not blind.** | **25-26** |
|  |  | **27** | Implications for practice, including the intended use and clinical role of the index test  **Discussed in discussion section.** | **22-29** |
|  | **OTHER INFORMATION** |  |  |  |
|  |  | **28** | Registration number and name of registry | **Not applicable** |
|  |  | **29** | Where the full study protocol can be accessed | **In manuscript** |
|  |  | **30** | Sources of funding and other support; role of funders  **This study was funded by Science and Engineering Research Board, India and Indian Council of Medical Research, India.** | **In submission form** |
|  |  |  |  |  |
