# Supplementary material for: Glycoproteins in circulating immune complexes are biomarkers of patients with Indian PKDL: A study from endemic districts of West Bengal, India
Source: PLoS One. 2018 Feb 8;13(2):e0192302. doi: 10.1371/journal.pone.0192302 (PMC5805291; doi:10.1371/journal.pone.0192302)
Supplement: S1 Fig — (DOCX) [file pone.0192302.s002.docx]

**S1 Fig: Flow diagram of the study subject**
